# Multifaceted intervention to enhance the screening and care of hospitalised malnourished children: study protocol for the PREDIRE cluster randomized controlled trial

**DOI:** 10.1186/1472-6963-13-107

**Published:** 2013-03-22

**Authors:** Sandrine Touzet, Antoine Duclos, Angélique Denis, Lioara Restier-Miron, Pauline Occelli, Stéphanie Polazzi, Daniel Betito, Guillaume Gamba, Fleur Cour-Andlauer, Cyrille Colin, Alain Lachaux, Noël Peretti

**Affiliations:** 1 Hospices Civils de Lyon, Pôle Information Médicale Évaluation Recherche, Lyon, F-69003, France; 2 Université de Lyon, EA Santé-Individu-Société 4129, Lyon, F-69002, France; 3 Hospices Civils de Lyon, Hôpital Femme Mère Enfant de Lyon, Bron, F-69677, France; 4 Hospices Civils de Lyon, Direction du Système d’Information et de l’Informatique, Bron, F-69500, France; 5 INSERM CIC201, Service de Pharmacologie Clinique, EPICIME, Hospices Civils de Lyon, Bron, F-69500, France; 6 Université de Lyon, INSERM U1060, CarMeN laboratory, Lyon, F-69008, France

**Keywords:** Malnourished children, Nutritional support team, Computerized clinical decision support system, Multifaceted intervention, Cluster randomized trial

## Abstract

**Background:**

Hospital malnutrition is an underestimated problem and as many as half of malnourished patients do not receive appropriate treatment. In order to extend the management of malnutrition in health care facilities, multidisciplinary teams focusing on clinical nutrition were established in France. The establishment of such teams within hospital facilities remains nonetheless difficult. We have consequently developed a multifaceted intervention coordinated by a Nutritional Support Team (NST). Our study aims to evaluate the impact of this multifaceted intervention coordinated by a NST, in adherence to recommended practices for the care of malnourished children, among health care workers of a paediatric university hospital.

**Methods/design:**

We carried out 1) a six-month observational phase focusing on the medical care procedures relative to malnourished children followed by 2) a cluster randomised controlled trial phase to evaluate the impact of a multidisciplinary nutrition team over an 18 month time frame.

Based on power analyses and assuming a conservative intracluster correlation coefficient, 1289 children were needed to detect a 25% difference in rates between the two groups of the cluster trial.

The implementation of our intervention was coordinated by the NST and had three major components: a) access to a computerised malnutrition screening system associated with an automatic alert system, b) an awareness campaign directed toward the health care workers and c) a leadership based strategy.

Main outcomes included the number of daily weighings during hospitalisation, the investigation of malnutrition etiology and the management of malnutrition by a dietician and/or the NST.

Due to the clustered nature of the data with children nested in departments, a generalized estimated equations approach will be used to analyse the impact of the multifaceted intervention on primary and secondary outcomes.

**Discussion:**

Our results will provide an overall response regarding the effectiveness of our multifaceted intervention and we should be able to suggest an organization and mode of operation of NST.

**Trial registration:**

ClinicalTrials.gov: NCT01081587.

## Background

The problem of hospital malnutrition has been identified only over the last thirty years or so. It is an issue that occurs frequently, with 15 to 30% of hospitalised children in industrialised nations qualifying as malnourished [1-3] and with nearly 50% at risk for malnutrition during their hospital stay [4]. Because it increases morbidity and mortality risk for the subjects affected (through issues such as infections, metabolic disorders, pressure ulcers and postoperative complications), malnutrition is potentially serious [5].

Hospital malnutrition is an underestimated problem. As many as half of malnourished children do not receive appropriate treatment for this issue, even in university hospital centres [6,7]. One of the main obstacles to the provision of quality care to malnourished children is their dispersal among a hospital’s various medical departments [8]. In addition, this care requires the coordinated involvement of physicians, dieticians, nurses, paediatric auxiliary nurses and physiotherapists.

The Committee on Nutrition of the European Society for Paediatric Gastroenterology, Hepatology and Nutrition (ESPGHAN) has issued recommendations to establish nutrition support teams in paediatric hospitals, to implement screening for nutritional risk, to identify patients who require nutritional support, to provide adequate nutritional management, to educate and train hospital staff, and to audit practice [9]. In order to extend the management of malnutrition in health care facilities, multidisciplinary teams focusing on clinical nutrition were established on an experimental basis in France starting in 2007 [10]. These multidisciplinary nutrition teams comprise physician nutrition specialists, nurses and dieticians. Such forms of organisation should facilitate the treatment of malnutrition in hospitals, involving clinical teams in systematic nutritional assessment that enables screening and leads to appropriate treatment [11]. The introduction of such teams should make it possible to reduce the prevalence of complications linked to malnutrition, improve the prescription of nutritional supplements and promote the dissemination of good practice, thereby leading to substantial economic savings [11].

The establishment of such teams within hospital facilities remains nonetheless difficult. In addition, the Nutritional Support Team (NST) is not always addressed appropriately, and an intervention strategy is required. We have consequently developed a multifaceted intervention that was coordinated by the NST and that aimed to:

– Raise awareness regarding the malnutrition issue,

– Train clinical teams regarding guidelines for good practice,

– Facilitate the screening of malnourished children through the use of an electronic alert system,

– Assist in decision making regarding the clinical teams’ care and treatment of malnourished children, with either a dietician or the NST enlisted to provide expertise,

– Coordinate nutritional care among several categories of health care professionals (nurses, auxiliaries, physicians and dieticians).

French guidelines for good practice relative to hospital malnutrition refer to a nutritional care algorithm that uses the children’s weight/height and height/age ratios [12]. The integration of this algorithm, as an automated decision support tool within the hospital data processing system, should improve the relevance of the multidisciplinary nutrition team’s interventions on hospitalised malnourished children [13].

This study aims to evaluate the impact of a multifaceted intervention (including electronic medical alerts) coordinated by a NST, in compliance with to recommended practices for the care of malnourished children, among health care workers of a paediatric university hospital.

## Methods/design

### Study design

Within a paediatric university hospital, we carried out 1) a six-month observational phase focusing on the medical care procedures relative to malnourished children followed by 2) a cluster randomised controlled trial phase to evaluate the impact of a multidisciplinary nutrition team over an 18 month time frame. This cluster randomised design was chosen because the multifaceted intervention was conducted at the practice level and outcomes were measured at the patient level.

### Participants

Our inclusion and exclusion criteria, at both cluster and patient levels, are presented in Table 1.

**Table 1:** Inclusion and exclusion criteria

**Population at cluster level**	
***Study units***	
*Inclusion criteria*	Medical and surgical units in a teaching hospital providing paediatric acute care
*Exclusion criteria*	Neonatal care units, intensive care units and emergency units
***Health care workers***	
*Inclusion criteria*	Any hospital staff involved in direct patient care (includes physicians, nurses, nursing assistants, physiotherapists and dieticians)
**Population at patient level**	
*Inclusion criteria*	Age 1 month to 18 years old
	Children recorded as malnourished based on their weight/height and height/age ratios upon hospital entrance, according to the Algoped nutritional risk score
*Exclusion criteria*	Age under one month, liver or kidney abnormalities, severe heart failure
	First weight recorded more than 2 days after admission, first height recorded more than 15 days after admission
	Stay less than 2 days

Head physicians of all eligible departments were contacted and invited to participate. All medical and surgical departments providing acute care in this paediatric university hospital participated in the trial (*i.e.*, 8 departments, 393 acute care beds (207 in medicine and 59 in surgery) performing 29 518 stays a year) except neonatal, intensive care and emergency units. Approximately 300 professionals were targeted (55 physicians and medical residents, 115 nurses, 12 dieticians, 100 paediatric auxiliary nurses and 27 physiotherapists).

Some nursing teams work across several departments. As a consequence, because our intervention targeted the practices of health care professionals, we grouped the relevant departments together into clusters in order to minimise contamination bias between departments. A total of six clusters were determined.

According to the French paediatric guidelines for the screening and treatment of malnutrition, the Algoped nutritional risk score was employed for assessing the nutritional status of every child based on weight and height measured at admission by health care workers [11]. Detection of malnourished children was based on the combined interpretation of two ratios. First, in order to detect acute malnutrition (stunting), the weight/height ratio (W/H) was calculated by dividing the child’s observed weight by the expected weight related to their observed height [14]. Second, in order to detect chronic malnutrition (wasting), the height/age ratio (H/A) was calculated by dividing the child’s observed height by the expected height related to their age. In cases of W/H > 90% and/or H/A > 94%, there was considered to be no malnutrition in the patient. In cases of W/H 80-90% and/or H/A 85-94%, there was moderate malnutrition, while W/H < 80% and/or H/A < 85% indicated severe malnutrition [15,16].

### The nutritional support team

The NST was composed of one paediatrician specialised in gastroenterology and clinical nutrition as well as two dieticians. This team was created one year prior to study implementation. The team usually intervenes at the request of the clinical teams in order to confirm the diagnosis of malnutrition in cases of doubt, to coordinate nutritional care and provide relevant advice, and if necessary to prescribe nutritional support (oral nutritional supplement, enteral or parenteral nutrition).

### Intervention

**Figure 1:**
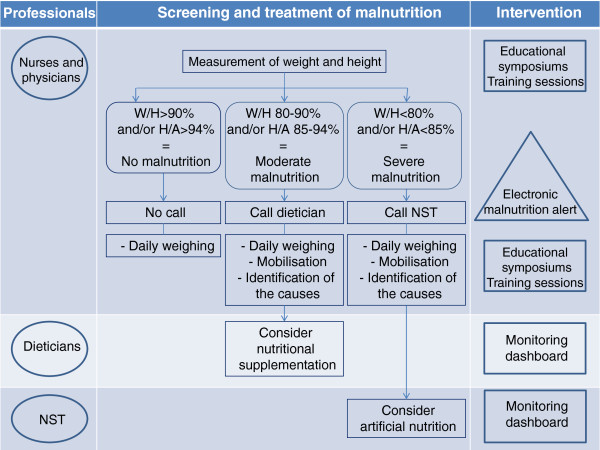
**The multifaceted intervention. **This figure presents the different components of the multifaceted intervention, as well as the professionals (target groups) and the relevant stages of nutritional treatment. NST: Nutritional Support Team.

The implementation of our multifaceted intervention was coordinated by the NST and pertains to the cluster level. It had three major components: a) access to a computerised malnutrition screening system and automatic alert system, b) an awareness campaign directed toward the health care workers and c) a leadership based strategy (see Figure 1).

#### Computerised tools

A computerised malnutrition screening system was made feasible through the nurses’ measurement of child’s height and weight at admission and the collection of these data in the IT system. Using this information in addition to the child's age and sex, the Algoped algorithm ran automatically, alerting physicians about the nutritional status of every child at the time when drugs were prescribed electronically. The message appeared in real time when the physician sought to prescribe a treatment, indicating “moderate malnutrition” or “severe malnutrition” based on the automatic calculation of the nutritional status of the child, in accordance with the calculation instructions of the Algoped algorithm:

– In the absence of malnutrition, no message appeared;

– In case of moderate malnutrition, the message indicated that the prescribing physician should make contact with the department dietician in order to decide what measures to take;

– In case of severe malnutrition, the message indicated that the prescribing physician should make contact with the NST in order to decide what kind of nutritional support to perform.

This algorithm was intended to facilitate the coordination of the treatment of malnutrition among members of the medical teams, the dieticians and the NST.

In addition, a monitoring dashboard was updated daily, providing the dieticians with the list of malnourished children in their department and allowing them to intervene independently of clinicians’ calls. The NST also had access to this dashboard.

#### Awareness campaign for health care workers

This awareness campaign consisted of 3 approaches: educational symposiums, training sessions and regular outreach visits to the various departments with the aim of informing health care workers about the frequency and severity of malnutrition among hospitalised children. Each approach was adapted to a specific professional category (physicians, dieticians, nurses and auxiliary nurses). In particular, the message aimed at nurses focused on appropriate screening, which requires systematic weight and height measurements. On the other hand, physicians and dieticians were made aware of evidence based care with which to comply, once they had been alerted about the child’s malnutrition status through the IT system. As well, the teams were trained to refer to the NST as appropriate, namely based on the degree of severity of the malnutrition observed.

In addition to this awareness campaign, case reviews were given by the NST during the initial months of the intervention in the various departments of the intervention arm (two case reviews per department). During these case reviews, the NST presented the files of malnourished children treated in the department where the review session was held. These case reviews involved the participation of physicians, dieticians, nurses and auxiliaries nurses.

#### Leadership based strategy

A leadership oriented strategy focused mainly on increasing the sensitivity of the computerised malnutrition screening system. To this end, the medical and nursing advisory boards of the hospital established the mandatory measurement of the weight and height of each hospitalised child, as well as the mandatory entry of these data into the IT system. In terms of intervention, the medical heads and nursing coordinators of each department were in charge of ensuring compliance with this recommendation. In addition, the NST was responsible for coordinating the dieticians across the hospital and for leading a periodic multidisciplinary meeting in order to analyse clinical incidents related to nutritional care and to facilitate the sharing of experience among attendees from various medical specialties.

### Nutritional support policy

Nutritional support provided by the department dietician and/or by the NST depended on the seriousness of nutritional status, the functionality of the digestive tract, the causal pathology and the prognosis. In case of moderate or severe malnutrition, daily weighing and investigation of malnutrition etiology were prescribed respectively by the department dietician or by the NST. Physiotherapy was prescribed when appropriate. Moreover, in cases of severe malnutrition, nutritional support was initiated as soon as possible, using the enteral or parenteral route, or both, according to the judgment of the NST.

### The control group

In the control arm, no specific intervention was implemented to improve the management of malnutrition (*i.e.*, with no access to the computerised malnutrition screening system, no educational campaign and no leadership based strategy) and the teams provided care as usual. In addition, as usual, the NST could be called for counselling at any time within the control arm.

### Outcomes and measurements

Primary outcomes were chosen for their ability to assess the quality of care within each cluster based on health care workers’ adherence to recommended practices for every malnourished child; they included the number of daily weighings during hospitalisation (total number of weighings divided by length of stay), the investigation of malnutrition etiology (a detailed analysis of malnutrition causes is present in the medical record) and the management of malnutrition by a dietician and/or the NST.

#### Secondary outcome measures

*For clinical practice*: Appropriate call-in of the NST;

*For clinical impact on children*:

Incidence of complications linked to nutritional status during the hospital stay;

Evolution of nutritional status during the hospital stay;

*For economic measures*: Mean length and cost of stay.

Data concerning primary and secondary outcome measures were available at the patient level and were extracted from the medical records by an independent research team. The research team received handouts and training to become familiar with data collection. Data relative to the length of stay and the cost of treatment were obtained from the hospital’s claims databases.

The same data were gathered for the observational study and for the interventional study.

In addition, we monitored the intervention process. The research team gathered information on the availability and usage of the information technology tools, on the actions carried out by the NST in the framework of the awareness campaign for health care workers, and on the actions conducted within the leadership based strategy.

### Sample size calculation and randomisation

On the basis of a consensus within the study group, we determined the absolute improvement of the primary outcome measure of 25% between the intervention condition and the control condition on the primary outcome measure. We calculated sample size with a method that takes into account the intracluster correlation coefficient (ICC), the number of events, the expected effect, and the power of the study [17].

Our trial was designed with a limited number of cluster units (6 cluster units) able to participate. Using an assumed ICC of 0.05, which is a conservative assumption [18], an expected cluster size of 200 children, a worst case control rate of 50%, a two-sided alpha level of 0.05 and a power of 80%, 1289 children were needed to detect a 25% difference in rates between the two groups.

Following the baseline observation phase, the six clusters were randomly assigned to the intervention condition or to the control condition.

### Blinding

Health care providers, participants and researchers were not blinded to group allocation.

## Ethical approval and informed consent

Approval for the study was obtained from the Sud Est III Institutional Review Board (study identifier: 2008–036 B) and from the French Data Protection Agency (CNIL).

As the promoter of this biomedical research, which falls within the scope of French Law n°2004-806 of 9 August 2004, the Hospices Civils de Lyon has acquired liability insurance coverage and stood as guarantors of the proper execution of the study.

Cluster leaders gave consent to the trial on behalf on the potential cluster members. Consent was sought before the observation phase and randomisation.

The parents of the children were informed of the study through an information sheet supplied with the patient welcome leaflet provided for each parent at the time their child is hospitalised. Parental written consent was not required in the context of this study [19]. In the event of a refusal to participate in the sharing of hospitalisation data for this study, the head of the hospital department was to record this information in the child’s medical file. Children could be withdrawn from sharing hospitalisation data but not from the intervention.

### Data analysis of the cluster randomised trial

All statistical analyses will be performed in Statistical Analysis System (SAS®) software version 9.2 (SAS Institute, Cary, NC). Analyses will be performed according to the intention-to-treat principle. All malnourished children screened in all clusters allocated to the intervention or control arm will be included in the analysis. In the event of multiple hospitalisations for the same patient, only the first stay of the study period will be retained.

Baseline characteristics will be summarised by pre- and post-intervention period and treatment group using frequency (percentage) for categorical variables and means (standard deviation) for continuous variables.

Clustering will be taken into account: a generalized estimated equations (GEE) approach will be used to analyse the impact of the multifaceted intervention on primary and secondary outcomes [20]. A GEE Poisson regression model will be performed for count data (as number of daily weighings) and GEE logistic regression models for binary variables (as investigation of malnutrition etiology, management by dietician, nutritional supplementation, complication occurrence during hospitalisation). The paediatric departments will be entered as the clustering variable into each model. For the GEE Poisson, the length of stay will be used as an offset. An exchangeable correlation matrix will be specified to account for potential within-cluster homogeneity in outcomes: all pairwise correlations among children within departments are the same. Robust estimates of the standard errors will be obtained which ensures, provided the model is correct, that the estimates are consistent even if the working correlation matrix is not correct. The impact of the multifaceted intervention will be adjusted for individual level characteristics included age, severity of malnutrition, comorbidities occurrence, complications occurrence and length of hospital stay after controlling for the collinearity between covariables. A Wald statistic will be used to test the significance of each coefficient in the model. The intervention effect will be expressed as adjusted odds ratio with its 95% confidence interval. GEE regression models will be fit using the SAS PROC GENMOD. Corresponding overall intra cluster correlation (ICC) coefficient was also estimated from GEE method using the CORRW option from the SAS PROC GENMOD.

For each arm, comparison of outcome changes between pre- and post-intervention periods will be computed using a GEE regression model, integrating the clustering of children by department. The period following intervention start will be the predictor, while all the potential confounders listed above will be considered in the final model based on a propensity score weighting approach. Immediate change and quarterly trend in clinical practices following the implementation of intervention will be also estimated.

All statistical tests were two-sided with p-value less than 0.05 regarded as significant.

### Time frame

The six month observational study was carried out from September 2009 to February 2010. The six clusters were randomised in February 2010. Data from the randomised trial were gathered from March 2010 to August 2011 (18 months). The statistical treatment of these data is currently underway.

Health care workers’ access to the computerised malnutrition screening system was established at the beginning of the intervention study, in the clusters of the intervention arm. The awareness campaign for the health care workers and the leadership based strategy were conducted intensively during the first three months of the intervention study, in the clusters of the intervention arm.

A support phase was conducted throughout the entire intervention period: this included the training of medical residents, the issuing of reminders to the teams regarding the use of the algorithm, and the organisation of department meetings in order to discuss the cases of malnourished children.

## Discussion

### Discussion of study design

We chose to conduct a cluster randomised trial. This choice is justified with respect to the form of the intervention, which is carried out at the level of the clinical teams (through educational intervention and a computerised malnutrition alert system) and at the level of the dieticians (through a dashboard identifying malnourished children). The intervention was targeted at health care professionals with the aim of studying its impact on patient outcomes [21]. No intervention was performed at the patient level. Data were gathered at the patient level, which means that our analysis had to take two levels into account: the patient level and the cluster level.

Clusters were designed in order to carefully prevent healthcare workers form cross-ward contamination bias. Only a few large sized clusters were available for study (six clusters). This small number of clusters could result in a relatively high probability of chance imbalance between arms. Clusters from our single pool were randomly assigned to the intervention condition or to the control condition. This completely randomised design is most suited to trials in which large numbers of clusters are available for randomisation. We could have opted for a pre-stratification or matching according to baseline characteristics. We will take into account any differences in the characteristics of the children between the clusters within the statistical analysis, by adjusting the models according to the variables of interest.

We performed an observational baseline phase. For six months, prior to the randomised trial, we collected data pertaining to children presenting the inclusion criteria. This phase allowed us to better understand how the treatment of malnourished children was implemented in the departments participating in the study and to test the feasibility of child enrolment. It allowed us to enable calculation of the ICC of the clinical department data (which confirmed that we could retain our hypothesis of an ICC of 0.05). Moreover, it will allow additional statistical analyses comparing main outcome measures before and after implementation of the multifaceted intervention.

Due to the clustered nature of the data with children nested in departments, a GEE approach will be used to analyse the impact of the multifaceted intervention on primary and secondary outcomes [20]. The characteristics and the health status of children in the same departments may be correlated, thus violating the independence assumptions of traditional regression models. In cluster randomized trials, standard methods for statistical analysis do not apply otherwise it will lead to underpowered studies and it will tend to bias observed p-values downward [22]. The GEE approach is also preferable rather than the random-effect regression models when testing the effects of cluster-level covariates (like the multifaceted intervention which is our only cluster-level exposure variable) [23], and when studying small number of clusters per arm.

### Discussion of the intervention

The intervention was developed by a multidisciplinary group, including the research team and the NST. The content of the message is consistent with evidence-based nutritional guidelines. We sought to implement change and measure improvement over an 18-month period, which is a relatively long study period.

One of the challenges presented by this study resides in the setup of the intervention. We monitored the intervention process in order to establish the feasibility of our intervention and its possible potential widespread use [24]. Our intervention involved the following three categories of professionals involved in the treatment of malnourished children: the clinical teams (physicians, nurses, auxiliary nurses and physiotherapists), the dieticians of the clinical departments, and the NST. The NST coordinated the awareness campaign and the leadership based strategy aimed at the clinical teams and the dieticians of the various departments. A computerised decision support tool was made available to the clinical teams. The dieticians, as well as the NST, had access to a monitoring dashboard identifying malnourished children in order to intervene on their behalf without waiting for validation from the clinical team.

The intervention conducted is based on theoretical assumptions of educational (problem based learning) and leadership theories of the individual [25,26]. Our educational strategy focused on the training of healthcare workers to screen malnourished children and to call a dietician or the NST, depending on the severity of the malnutrition. We associated a decision support tool with these approaches. The algorithm upon which our computerised support tool is based meets a reproducible, validated standard of practice that complied with the national recommendations for the screening and treatment of malnutrition in hospitalised children [12]. Our automated decision support tool for the screening of malnourished children can be described as a Computer-based Clinical Decision Support System, associating alerting and reminding functions [27]. Its deliberately simple message [28] aims to guide the professional toward the department dietician or toward the NST as required. The effectiveness of such systems has been proven within various hospital organisations [29]. In the evaluation of an intervention comprising several components, the challenge lies in identifying what has an impact on the practices and behaviours of the practitioners [30]. Our results will provide an overall response regarding the effectiveness of our multifaceted intervention and we should be able to suggest an organization and mode of operation of NST.

## Abbreviations

NST: Nutritional support team; ICC: Intracluster correlation coefficient; H/A: Height/age ratio; W/H: Weight/height ratio.

## Competing interest

The authors declare that they have no competing interests.

## Authors’ contributions

All listed authors contributed to the conception and design of the study. All authors were involved in drafting the manuscript and read and approved the final manuscript.

## Pre-publication history

The pre-publication history for this paper can be accessed here:

http://www.biomedcentral.com/1472-6963/13/107/prepub
